# Pediatric Chronic Kidney Disease: Mind the Gap Between Reality and Expectations

**DOI:** 10.3390/children12050614

**Published:** 2025-05-08

**Authors:** Chien-Ning Hsu, Pei-Chen Lu, Wei-Ting Liao, You-Lin Tain

**Affiliations:** 1 Department of Pharmacy, Kaohsiung Chang Gung Memorial Hospital, Kaohsiung 833, Taiwan; cnhsu@cgmh.org.tw; 2 School of Pharmacy, Kaohsiung Medical University, Kaohsiung 807, Taiwan; 3 Division of Pediatric Nephrology, Kaohsiung Chang Gung Memorial Hospital, Kaohsiung 833, Taiwan; latina@cgmh.org.tw (P.-C.L.); winona0409@cgmh.org.tw (W.-T.L.); 4 College of Medicine, Chang Gung University, Taoyuan 333, Taiwan; 5 Department of Pediatrics, Kaohsiung Municipal Ta-Tung Hospital, Kaohsiung 801, Taiwan

**Keywords:** chronic kidney disease, guideline, developmental origin of health and disease (DOHaD), congenital anomalies of the kidney and urinary tract (CAKUT), children, biomarker, health-related quality of life

## Abstract

Pediatric chronic kidney disease (CKD) is a growing concern that often originates early in life, yet significant challenges remain in translating clinical guidelines into real-world practice. World Kidney Day 2025 highlights the importance of early detection, but the three levels of preventive strategies commonly recommended for adults may not be directly applicable to children. Unlike adult CKD, primary prevention in pediatrics should focus on prenatal, neonatal, and early-life factors such as congenital anomalies of the kidney and urinary tract (CAKUT), preterm birth, maternal health, and environmental exposures. Secondary prevention, involving early detection through screening, is crucial, yet the effectiveness of mass urinary screening in children remains a subject of global debate. Several key challenges persist, including the accurate estimation of glomerular filtration rate (eGFR), consistent definition and diagnosis of pediatric hypertension, identification of reliable biomarkers, and targeted screening in specific pediatric populations. Although clear guidelines exist to manage CKD progression and enhance quality of life, a critical gap remains between what is known and what is practiced. Closing this gap requires robust evidence to inform best practices, improve health-related quality of life, and advance pediatric kidney replacement therapies. To protect and improve kidney health for every child worldwide, these challenges must be acknowledged, and sustainable, evidence-based solutions must be developed and implemented without further delay.

## 1. Introduction

Chronic kidney disease (CKD) poses a significant global health challenge, currently impacting approximately one in ten adults worldwide [1]. Ranked as the third-fastest-growing cause of death, CKD is expected to become the fifth leading cause of mortality by 2040 [2]. Importantly, CKD is preventable, and its progression to end-stage kidney disease (ESKD) can be delayed with timely interventions, especially when implemented early. Recognizing this, the global kidney health community emphasizes the need to identify children at higher risk for CKD throughout their lifetimes and develop targeted prevention strategies [3]. Research has extensively examined risk factors for CKD development during prenatal, neonatal, and early childhood stages, as adult CKD often originates in early life [4]. This concept is known as the developmental origins of health and disease (DOHaD) [5,6].

Despite strong evidence-based clinical guidelines for CKD prevention [7], adherence remains suboptimal [8,9]. In pediatric populations, CKD management requires special consideration due to unique etiologies, growth and developmental factors, kidney maturation, and long-term risk assessment [7]. A lifespan approach incorporating the DOHaD framework is therefore essential [7,10]. However, translating CKD management guidelines to children and adolescents requires careful adaptation. Understanding the gaps between real-world practice and guideline expectations in pediatric CKD is critical.

**Figure 1:**
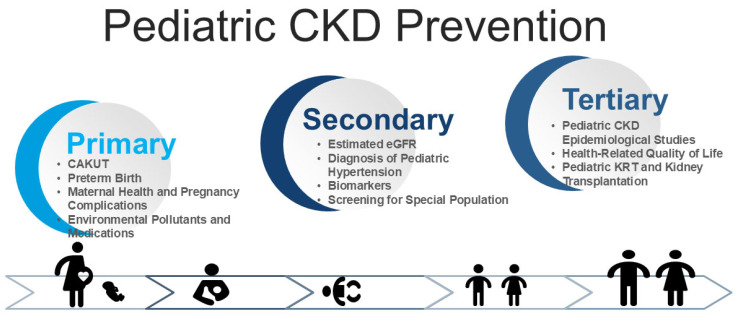
Conceptual framework illustrating the integration of primary, secondary, and tertiary prevention across the life course, with an emphasis on key clinical challenges in the management of pediatric chronic kidney disease (CKD).

CKD prevention follows a three-tiered approach [11]: (1) primary prevention, which aims to prevent disease onset; (2) secondary prevention, which focuses on early detection and intervention to slow progression; and (3) tertiary prevention, which manages established CKD to reduce complications. The World Kidney Day 2025 initiative highlights that primary and secondary prevention strategies can leverage the “8 Golden Rules” for kidney health promotion [12]. While numerous kidney protection strategies exist, their direct applicability to pediatric CKD remains a challenge [13]. This review aims to address these critical gaps to enhance clinical practices across the lifespan, ultimately reducing global CKD risk. Figure 1 illustrates the three levels of prevention—primary, secondary, and tertiary—within a life course approach, highlighting key challenges in pediatric CKD.

## 2. Materials and Methods

This narrative review aims to consolidate recent findings and highlight the gaps between knowledge and clinical practices in pediatric CKD. We conducted a comprehensive literature review by identifying relevant studies published in scientific databases such as the Cochrane Library, MEDLINE, and Embase. Our research includes clinical studies, observational studies, clinical trials, guidelines, and reviews published from January 2000 to March 2024, focusing on full-text articles written in English. We specifically included studies addressing management and prevention strategies in pediatric CKD while excluding similar research to highlight the most representative findings.

The search utilized relevant keywords, including “chronic kidney disease”, “congenital anomalies of the kidney and urinary tract”, “cardiovascular disease”, “arterial stiffness”, “atherosclerosis”, “endothelial dysfunction”, “hypertension”, “blood pressure”, “ambulatory blood pressure monitoring”, “left ventricular mass index”, “biomarker”, “omics”, “children”, “pediatric”, “childhood”, “DOHaD”, “uremic toxins”, “risk factors”, “screening”, “glomerular filtration rate”, “health-related quality of life”, “kidney replacement therapy”, and “kidney transplantation”. Additionally, we reviewed reference lists to identify other relevant sources.

## 3. Challenges in Primary Prevention

The key difference in CKD primary prevention between children and adults lies in the timing of disease onset, which determines the modifiable risk factors targeted in prevention strategies. In adults, CKD prevention primarily focuses on lifestyle-related risk factors, such as diet, physical activity, alcohol consumption, and tobacco smoking [14]. The eight golden rules for primary prevention of adult CKD include maintaining a balanced and nutritious diet, ensuring adequate hydration, engaging in regular physical activity, monitoring and managing blood pressure (BP), regulating blood glucose levels, avoiding nicotine use, refraining from the routine use of nonsteroidal anti-inflammatory drugs (NSAIDs), and conducting targeted screening in individuals with known risk factors [12]. Notably, five of these preventive measures align with the “Life’s Essential 8” guidelines for promoting and sustaining cardiovascular health, which additionally emphasize maintaining a healthy weight, ensuring sufficient sleep, and managing blood lipid levels [15]. While some of these principles are applicable to children and adolescents, others—such as avoiding nicotine, regular use of NSAIDs, and physical activity—do not apply to infants and fetuses.

In contrast, pediatric CKD primary prevention emphasizes prenatal, neonatal, and early-life factors, including congenital anomalies of the kidney and urinary tract (CAKUT), preterm birth, maternal health, pregnancy complications, and early-life exposure to environmental pollutants and medications [3,16,17,18].

### 3.1. CAKUT

CAKUT are the major causes of pediatric CKD [19]. Human kidney development begins around week 3 of gestation and continues until about week 36 [20]. Disruptions during this period can impair nephrogenesis, reducing nephron number and resulting in clinical manifestations of CAKUT [19]. Despite the identification of several candidate genes, over 80% of CAKUT cases cannot be attributed to a single genetic cause. This indicates that genetic, epigenetic, and environmental factors likely contribute to the condition [19]. A case–control study involving more than 1.6 million infants identified several risk factors for CAKUT, including premature birth, low birth weight (LBW), maternal gestational diabetes, maternal thalassemia/hemochromatosis, polyhydramnios or oligohydramnios, first parity, and male sex [21].

A reduced nephron number raises glomerular pressure, triggering hyperfiltration and compensatory glomerular and tubular hypertrophy, which accelerates ongoing nephron loss [22]. Given these complexities, there is an urgent need for long-term prospective studies on children with CAKUT, combined with genomic analysis and a thorough assessment of in utero environmental risk factors. Such studies would enhance our understanding of the pathogenesis of CAKUT and aid in developing effective preventive strategies.

An additional challenge is the inability to determine nephron number in vivo. While progress has been made using cationized ferritin as a targeted MRI contrast agent to measure nephron numbers in both human and rat kidneys [23,24], further validation of non-invasive methods to assess nephron endowment in vivo remains a priority.

### 3.2. Preterm Birth

Most nephron formation occurs during the third trimester—a period often interrupted by preterm birth, defined as delivery before 37 weeks of gestation [25]. Preterm birth is frequently linked to low birth weight (LBW, <2500 g), and while distinct, the two conditions often overlap due to shared risk factors. Premature birth has been consistently associated with adverse kidney outcomes spanning from infancy to adulthood, including reduced kidney volume and function, elevated BP, microalbuminuria, and increased risk of CKD [26,27,28,29,30]. Contributing factors such as maternal malnutrition, illness, pregnancy complications, exposure to environmental chemicals, and certain medications can influence both preterm birth and kidney development [31]. Proposed mechanisms include oxidative stress and glucocorticoid exposure during pregnancy [32,33], though high-quality, long-term studies and clinical guidelines remain limited, leaving pediatricians with few evidence-based strategies for managing these risks.

Additionally, preterm infants face numerous postnatal challenges that may further elevate their risk of developing CKD. These challenges include both endogenous vulnerabilities and iatrogenic factors encountered during neonatal intensive care. Collectively, they represent a “second hit” to the already compromised kidney development of preterm infants. Key contributors include episodes of acute kidney injury (AKI); exposure to nephrotoxic medications such as aminoglycosides and NSAIDs; and suboptimal nutrition that fails to meet the unique demands of preterm growth. Furthermore, rapid postnatal weight gain or catch-up growth, though often necessary to reduce immediate morbidity, may impose additional stress on the kidneys by exacerbating glomerular hyperfiltration and promoting metabolic abnormalities.

AKI is a significant concern for neonates in the neonatal intensive care unit, with a systemic review indicating a 25% AKI rate among preterm infants, which is linked to high mortality [34,35]. Major risk factors include LBW, sepsis, low Apgar scores, mechanical ventilation, patent ductus arteriosus (PDA), vasoactive drugs, NSAIDs, and nephrotoxic antibiotics [36]. Premature kidneys, especially in LBW infants, are particularly vulnerable to injury from stressors like sepsis and mechanical ventilation, which can exacerbate renal damage [37,38]. Additionally, complications such as necrotizing enterocolitis can further reduce blood flow and contribute to AKI [36]. Nephrotoxic medications, including antibiotics and diuretics, are commonly used in the NICU, with over 80% of neonates receiving at least one potentially harmful drug [39,40]. Factors like age and drug dosage influence nephrotoxicity, and lower gestational age can impair drug clearance, increasing toxicity risk [41]. Reducing exposure to nephrotoxic drugs is crucial for decreasing AKI rates and preventing CKD later in life.

Moreover, preterm infants have higher nutritional needs than term infants, making them more susceptible to deficiencies that can hinder growth and development [42]. The DOHaD research explores hypotheses like the thrifty phenotype and catch-up growth [43,44], linking early nutritional imbalance to later chronic diseases, including CKD. Rapid weight gain in preterm infants increases the risk of metabolic syndrome, elevated BP, and insulin resistance from early childhood [45]. Despite existing nutrition guidelines, a lack of consensus leads to variability in clinical practice. Monitoring growth in the first two years is crucial, as early patterns may impact long-term cardiometabolic health and contribute to subclinical kidney damage [46,47]. Emerging research highlights the interconnected nature of cardiovascular, kidney, and metabolic disorders, with CKD recognized as a key component of the cardiovascular–kidney–metabolic syndrome (CKMS) [48,49]. These findings underscore the importance of early, targeted interventions to address modifiable risk factors and potentially slow the progression toward CKD initiated by impaired nephrogenesis.

### 3.3. Maternal Health and Pregnancy Complications

CKD is associated with an increased risk of adverse pregnancy and fetal outcomes, such as preeclampsia, preterm birth, small-for-gestational-age (SGA) infants, and accelerated loss of maternal kidney function [50,51,52]. Guidelines for the care of pregnant women with CKD address preconception counseling, risk assessment, and specialized prenatal care and screening, as well as specific treatment options [53,54,55]. However, current recommendations primarily focus on maternal health and immediate pregnancy outcomes, leaving a need for further research and guidelines that address the prevention and management of kidney health in children born to mothers with CKD.

In addition to CKD, other maternal illnesses and obstetric complications—such as diabetes [56], preeclampsia [57], maternal hypoxia [58], reduced uterine perfusion [59], and inflammation [60]—have been shown to induce kidney programming, resulting in kidney disease in offspring in animal models. Human studies indicate that adults born to mothers with gestational diabetes have a higher risk of CAKUT and kidney disease [61,62]. A meta-analysis further supports that maternal obesity negatively affects renal programming in offspring, increasing the risk of kidney disease in adulthood [63]. Therefore, effectively managing maternal health conditions and addressing pregnancy complications are crucial for promoting normal delivery and healthy fetal development, which can significantly reduce the risk of CKD in offspring.

Moreover, substance abuse significantly impacts maternal health, with 6–16% of pregnant women in the U.S. using alcohol, tobacco, or illicit drugs [64]. A study found that maternal alcohol exposure harms kidney function in overweight and obese children in a dose-dependent manner [65], while another linked maternal alcohol use to mild CKD in offspring by age 30 [66]. In a rat model, maternal ethanol exposure resulted in reduced nephron numbers and kidney function in adult offspring, likely due to inhibited ureteric branching morphogenesis [67]. Maternal smoking during pregnancy is linked to reduced fetal and infant kidney volume, with animal studies showing that nicotine exposure harms kidney development, potentially leading to CKD in offspring [68,69]. While illicit drug use increases CKD risk, its impact on offspring renal outcomes is still unclear [70]. Preventive strategies should focus on helping pregnant women quit smoking and drinking.

### 3.4. Environmental Pollutants and Medications

Environmental pollutants and medications can disrupt kidney development, leading to low nephron numbers and CAKUT [17,71,72]. In a rat model, maternal exposure to di-2-ethylhexylphthalate (DEHP) resulted in reduced kidney function and hypertension in offspring, likely due to gene dysregulation during nephrogenesis [73]. Other endocrine-disrupting chemicals (EDCs) like bisphenol A (BPA), TCDD, and phthalates also impair kidney development by interfering with hormone signaling [74,75,76]. Emerging evidence suggests that fetal EDC exposure may increase CKD risk later in life, underscoring the need for further research and screening as part of preventive CKD strategies.

Additionally, various medications administered to pregnant women may affect kidney development and cause CAKUT [72]. These medications cover Adriamycin, anti-epileptic drugs, aminoglycosides, renin–angiotensin system (RAS) blockers, NSAIDs, dexamethasone, furosemide, cyclosporine, and cyclophosphamide. For instance, a study found that prenatal exposure to systemic glucocorticoids significantly heightened the risk of childhood CKD, particularly in cases of preterm birth and when higher doses were administered [77]. Animal studies support these findings, demonstrating that glucocorticoid exposure during nephrogenesis adversely affects kidney outcomes in adult offspring [78]. As previously mentioned, NSAIDs pose a risk for AKI in premature infants. Furthermore, a recent study revealed that pregnant women exposed to NSAIDs were significantly associated with CKD in their child, especially during the second and third trimesters with specific NSAIDs [79]. Although recommendations exist to avoid nephrotoxic agents in pregnant women, real-world practices often fall short of these guidelines.

## 4. Challenges in Secondary Prevention

Secondary prevention aims to detect CKD early and slow its progression to prevent complications. Early screening allows for interventions that can modify risk factors and slow kidney function decline. A systematic review found that screening high-risk populations for CKD is cost-effective, while evidence for screening the general population is limited [80]. Mass urinary screening is commonly implemented in Asian countries such as Japan [81], Taiwan [82], and Singapore [83]. Nevertheless, there remains significant global debate about its effectiveness, especially in Western nations [84], where there is no consensus on its advantages for the general pediatric population. While urinary screening is viewed as cost-effective, its utility as a screening tool is often questioned [85].

Early identification of CKD offers significant public health benefits, but many countries still lack adequate surveillance systems to effectively monitor the condition [86]. According to KDIGO 2024, the initial screening tests for CKD should include urine dipstick for proteinuria, urine albumin-to-creatinine ratio (ACR), urine protein-to-creatinine ratio (PCR), and estimated glomerular filtration rate (eGFR) using serum creatinine (sCr) and cystatin C, BP measurement, blood glucose estimation, and height and weight measurement for BMI calculation [7]. However, not all recommendations can be easily applied to the pediatric population.

### 4.1. Estimated GFR

One important consideration outlined in the guidelines is the eGFR in children using validated equations that have been developed or validated in comparable populations [7]. In this context, many children and adolescents worldwide cannot calculate their eGFR due to a lack of appropriate equations. While the gold standard for measuring GFR uses inulin clearance, it is invasive and impractical in children [87]. Instead, sCr is commonly used to estimate GFR, as it is easy to measure and reflects filtration efficiency.

The original bedside Schwartz equation, developed in 1967, estimates GFR as eGFR = k × body height (BH)/SCr, with k varying by age [88]. In 2009, Schwartz revised this to the Schwartz IDMS equation: eGFR = 0.413 × BH/SCr, based on data from the Chronic Kidney Disease in Children (CKiD) study, suitable for children aged 1 to 16 years [89]. Although this formula is widely accepted for its simplicity and reasonable accuracy, it requires validation for application in other ethnic populations. Subsequently, Pottel et al. introduced a height-independent equation for children aged 1–14 years: eGFR = 107.3/(SCr/Q), where Q is the median sCr for healthy children of a specific age [90,91]. This equation was later extended to adolescents and young adults using Q-age polynomials and could also utilize Q-values based on height [92]. Over the next decade, Pottel et al. adapted their pediatric equation for adults, creating the first EKFC equation spanning the full age range to bridge pediatric and adult nephrology care [93]. However, local reference ranges for sCr are needed for optimal use, and future research should address eGFR differences between boys and girls or among ethnicities with normal kidney function [87].

### 4.2. Diagnosis of Pediatric Hypertension

Based on the KDIGO guidelines [7], hypertension in children with CKD is diagnosed using 24 h ambulatory blood pressure monitoring (ABPM). The recommended treatment target is to lower the 24 h mean arterial pressure (MAP) to the 50th percentile for age, sex, and height. However, several challenges persist in clinical setting [94], especially in children with CKD [95].

Currently, the prevalence and significance of pediatric hypertension all over the world cannot be accurately assessed due to the lack of a standardized international definition [96]. Pediatric hypertension guidelines have evolved since their inception in 1977, with the Fourth Report (2004) defining hypertension based on BP percentiles for age, sex, and height, requiring confirmation of BP readings ≥ 95th percentile across three visits [97]. Subsequent guidelines refined thresholds to align with adult standards [98,99], such as the European Society of Hypertension’s fixed 140/90 mmHg threshold for adolescents aged 16+ (2016) and the American Academy of Pediatrics’ simplified 130/80 mmHg threshold from age 13 (2017), which excluded overweight children from reference populations, increasing prevalence estimates. Hypertension Canada introduced outcome-based thresholds (130/85 mmHg for ages 12–17; 120/80 mmHg for ages 6–11) in 2020 [100]. Asian guidelines vary [101,102], with China’s formula-based screening method (2018) and Japan’s age- and sex-specific thresholds (2019). These variations in diagnostic criteria can result in differing clinical diagnoses across populations.

Both 24 h ABPM and home blood pressure (HBP) measurements are essential tools for diagnosing pediatric hypertension. However, the global use of ABPM remains limited due to high costs and limited accessibility, despite its key role in detecting white-coat and masked hypertension, particularly in children with CKD. HBP monitoring shows promise, demonstrating approximately 80% agreement with ABPM, but its use is constrained by the lack of validated devices for young children [103]. Clinic BP measurements alone often fail to provide accurate diagnoses, with studies showing that nearly half of referred children are found to have white-coat hypertension. There is a clear need for further research to develop cost-effective alternatives to ABPM and to better define the role of HBP in screening. Optimizing home monitoring could help reduce reliance on ABPM, especially in resource-limited settings.

Significant gaps in knowledge remain regarding the treatment of pediatric hypertension, with limited data to establish clear therapeutic goals for both nonpharmacologic and pharmacologic interventions [104]. While the KDIGO guidelines recommend targeting a 50th percentile 24 h MAP [7], other guidelines, such as the 2023 ESH, suggest a target office blood pressure of <140/90 mmHg for adolescents, leading to variability in treatment approaches.

The 2017 AAP guidelines recommend starting with lifestyle modifications—such as dietary changes and increased physical activity—and progressing to pharmacologic therapy if BP remains elevated or if left ventricular hypertrophy (LVH) is detected. Common pharmacologic options include RAS blockers, calcium channel blockers, and thiazide diuretics. These medications are generally safe for short-term use, though long-term outcome data are lacking [105]. FDA-approved antihypertensive drugs for children are primarily limited to those aged 6 years and older, with fewer options available for younger children and neonates. Accordingly, prospective trials are needed to determine whether lowering BP in youth reduces the long-term risks of adult hypertension, target organ damage, and cardiovascular disease in children with CKD [96].

### 4.3. Biomarkers

The use of biomarkers to detect CKD earlier on and improve children’s prognoses remains an unmet medical need. While numerous blood and urine biomarkers have been identified for adult CKD [106,107], research on pediatric-specific biomarkers is limited [108,109]. In the CKiD cohort study, plasma kidney injury molecule-1 (KIM-1), fibroblast growth factor-23 (FGF23), tumor necrosis factor receptor-1 (TNFR1), and TNFR2, as well as urine epidermal growth factor (EGF), KIM-1, and monocyte chemoattractant protein-1 (MCP-1), are independently linked to CKD progression [109]. However, larger studies with standardized methodologies are needed to validate these potential biomarkers and effectively integrate them into clinical practice for early diagnosis or detecting disease progression.

While promising biomarkers for CV risk have been identified in adults, efforts in pediatric CKD remain limited. Children are uniquely affected by ongoing kidney development, which influences CKD progression and management. Given the rarity of CV events in youth, early and aggressive prevention strategies may offer significant long-term benefits. In children with CKD, the pre-dialysis stage presents a critical window to identify risk factors and implement interventions to prevent future CVD.

Endothelial dysfunction, a key mechanism linking CKD to increased CV risk, involves reduced nitric oxide (NO) production. These NO-related biomarkers offer potential for early detection and management of CKD-related cardiovascular complications in pediatric populations [110]. Additionally, omics technologies have advanced the understanding of CKD by identifying biomarkers that reflect pathophysiological processes and CV risk [111]. In pediatric CKD, genomic studies like PediGFR have identified single-nucleotide polymorphisms (SNPs) linked to CKD, while transcriptomic analyses revealed differentially expressed genes in diseased kidney tissues [112]. Proteomic approaches have identified biomarkers like ApoC-II, CFH-related proteins, and plasma factor 4 associated with hypertension and LVH [113,114,115]. Metabolomic studies have highlighted metabolites such as sphingosine-1-phosphate and trimethylamine-N-oxide (TMAO) linked to CKD progression and CV risk [116,117,118]. Despite these advancements, the clinical application of omics biomarkers in pediatric CKD remains limited, necessitating further validation and integration into precision medicine for improved CV risk prediction and management.

### 4.4. Screening for Special Population

For preterm infants, essential screening services play a critical role in early detection and management of potential CKD-related complications. These services include antenatal screenings to identify any prenatal risks, renal ultrasounds to assess kidney structure and function, eGFR assessments to monitor kidney filtration capacity, BP monitoring to detect signs of hypertension or other cardiovascular issues, and genetic counseling to understand potential hereditary risks or conditions that could affect kidney development [31]. While several biomarkers for AKI have been investigated, none have yet been sufficiently validated, particularly in the vulnerable population of preterm neonates, where early detection is vital for minimizing long-term damage. Additionally, there is a pressing need for a reliable, non-invasive method to accurately estimate nephron number or “nephron endorsement”, a key factor for assessing kidney development and function. This tool would be invaluable not only for clinical practice but also for research, offering a more accurate and accessible way to monitor kidney health and better guide interventions in preterm infants.

A newly recognized at-risk population is those with CKMS. The American Heart Association has recently introduced the term CKMS to highlight the complex interplay between CKD and other adverse medical conditions [48,49]. CKMS is classified into four stages, reflecting varying degrees of severity and progression within its spectrum [119]. The syndrome’s main components emerge at different stages, influencing both its progression and impact. While CKMS is estimated to affect approximately 90% of adults in the United States [120], its prevalence and impact in the pediatric population remain unclear. Although holistic management—addressing the syndrome as a whole rather than treating individual conditions in isolation—is recommended for optimal care [48,49], comprehensive guidelines for pediatric CKMS have yet to be established. Early screening and recognition of CKD in children and adolescents with CKMS should be considered to enable timely intervention and improve outcomes.

## 5. Challenges in Tertiary Prevention

In children with advanced CKD, effective management of uremia and associated comorbidities—such as anemia, mineral and bone disorders, and CVD—is a high priority. These efforts are crucial for slowing progression to ESKD and improving overall quality of life [11]. Unlike adults, children face unique and serious complications, including growth and developmental delays, as well as urological abnormalities [121,122]. Once kidney failure occurs, long-term kidney replacement therapy (KRT), including kidney transplantation, becomes necessary and often extends throughout life. Therefore, comprehensive registries and cohort studies that capture detailed epidemiological data on pediatric CKD are essential for guiding improved patient care. Moreover, greater attention should be given to health-related quality of life across all CKD stages in children. Despite advancements, KRT and kidney transplantation continue to face significant challenges, highlighting the need for more robust evidence to inform best practices.

### 5.1. Pediatric CKD Epidemiological Studies

A previous review identified 24 registries and cohort studies on pediatric chronic kidney disease (CKD) from across the globe [123]. However, the classification of CKD, selection criteria, target age groups, and disease severity vary significantly between studies. Some large nationwide or international registries, such as the United States Renal Data System (USRDS), the Australia and New Zealand Dialysis and Transplant Registry (ANZDATA), and the European Renal Association—European Dialysis and Transplant Association (ERA-EDTA) registry, encompass all age groups but are not specifically focused on pediatrics. In contrast, most pediatric CKD cohorts are relatively small in size and have limited follow-up data.

The CKiD cohort, launched in 2003, is currently the largest pediatric CKD study, enrolling over 1000 participants to better understand CKD progression, associated comorbidities, and outcomes [124]. Another significant pediatric CKD cohort is the prospective Cardiovascular Comorbidity in Children with Chronic Kidney Disease (4C) study, which followed 704 children with stage 3–5 CKD from 12 European countries between 2010 and 2018 [125]. Since many clinical guideline recommendations are based on data from the CKiD study, it is important to note that, while it is a multicenter study conducted across the United States and Canada, it lacks international comparisons. Additionally, racial disparities in outcomes are multifactorial and may limit the generalizability of these guidelines to other countries. Given the diverse social, economic, ethnic, and religious contexts, as well as variations in insurance systems and medical resources across regions, there is a critical need for more evidence from pediatric CKD studies, particularly in Asia, Latin America, and Africa.

### 5.2. Health-Related Quality of Life

According to the 2024 KDIGO guideline, health-related quality of life (HRQOL) assessment in pediatric CKD should be an integral part of holistic care, although the guideline does not specify a single preferred tool [7]. It emphasizes individualized, comprehensive care that includes monitoring disease burden and complications affecting physical, emotional, and social well-being in children and young adults with CKD. The Pediatric Quality of Life Inventory (PedsQL) for children aged 3–18 years on physical, emotional, social, and school functioning has been extensively applied in children with CKD, showing sensitivity to differences by disease severity and treatment modality [126,127]. However, comparisons across different populations in the existing literature using PedsQL have been qualitative, with no statistical analyses performed.

The EQ-5D-Y (EuroQol-5 Dimension-Youth version) is a generic preference-based measure of HRQOL for children aged 7–18 years, which means it allows for health utility (quality-adjusted life year, QALY) scoring applied in health economics. EQ-5D-Y has also been studied in children and adolescents with mild-to-moderate CKD [128,129,130]. The visual analog scale (VAS) of EQ-5D-Y showed moderate correlations with the emotional and social domains of PedsQL, although convergent validity between the two instruments was limited in some dimensions of health state. The level-sum score (LSS) of EQ-5D-Y demonstrated greater sensitivity to changes in comorbidity status than the VAS in children with CKD. Choosing an HRQOL tool depends on the target population and purpose. EQ-5D-Y is brief, utility-based, and ideal for surveillance and economic evaluation, while PedsQL offers detailed condition-specific insights for clinical and research settings.

### 5.3. Pediatric KRT and Kidney Transplantation

Approximately 80% of all pediatric patients receiving KRT reside in Europe, North America, or Japan, where nearly all children with ESKD have access to treatment [131]. In contrast, KRT remains scarce or unavailable in many low- and middle-income countries due to limited financial resources and a shortage of trained healthcare professionals [132]. This disparity highlights a significant gap between clinical guideline recommendations and real-world practice.

For dialysis, especially in neonates and small children, technical difficulties such as establishing reliable vascular access, managing fluid and electrolyte balance, and preventing infections are significant hurdles. Dialysis in infants is complicated by poor nutritional status, growth failure, and bone disease, requiring careful nutritional and metabolic management. Hemodialysis is limited by the availability of machines adapted for small children and the risks associated with central venous catheters [131,132].

Pediatric kidney transplantation also faces several major challenges, including technical difficulties in very young or small children due to size mismatches and complex surgeries. Other concerns include managing growth and developmental issues, as well as minimizing long-term immunosuppressive side effects [133,134,135]. Vascular complications, post-transplant infections, and urinary problems are common, while the limited availability of donor organs and the negative effects of prolonged dialysis further complicate care. Additionally, there is no consensus on the optimal timing for transplantation, and most pediatric recipients will require re-transplantation during their lifetime. Lack of awareness among patients and donors, along with resource limitations in some regions, also restrict access to transplantation. Addressing these challenges necessitates a multidisciplinary approach aimed at improving surgical outcomes, long-term graft survival, and overall quality of life. Bridging the gap between clinical guidelines and real-world practice in pediatric CKD is critical to improving care and outcomes for these patients. These key gaps between guidelines and real-world practices are summarized in Table 1.

## 6. Conclusions and Future Perspectives

The major points of World Kidney Day 2025 center on the theme “Are Your Kidneys OK? Detect Early, Protect Kidney Health”, emphasizing the vital importance of early detection and intervention to prevent kidney disease and its complications. While early detection is universally critical, screening strategies and identification of at-risk populations in children differ significantly from those in adults. In pediatric CKD, there are notable gaps between guideline recommendations and real-world practice, particularly regarding the implementation of effective screening and prevention strategies. Persistent challenges include low public awareness, inadequate screening programs, and insufficient healthcare infrastructure—issues that are especially pronounced in low-income regions. Policymakers often underestimate the economic and societal impact of CKD, resulting in limited funding and action. We, therefore, call for coordinated collaboration among pediatricians, healthcare professionals, allied health workers, researchers, and policymakers to bridge these gaps and foster patient-centered, globally informed approaches to pediatric CKD prevention and care, ultimately aiming to improve kidney health outcomes worldwide.

## Figures and Tables

**Table 1:** Summary of key gaps across three levels of prevention.

**Primary Prevention**
Long-Term Prospective Studies on CAKUT
Non-Invasive Methods for Nephron Number Assessment
High-Quality, Long-Term Studies on Preterm Birth and Kidney Outcomes
Inconsistencies in Nutritional Guidelines for Preterm Infants
Research on Maternal Health and Offspring Kidney Outcomes
Impact of Substance Abuse on Kidney Health
Research on Environmental Pollutants and Kidney Health
Screening and Prevention for Maternal Medication on Offspring CKD Risks
**Secondary Prevention**
Inadequate Pediatric-Specific eGFR Equations
Lack of Standardized International Definition for Pediatric Hypertension
Limited Global Use and Accessibility of 24-Hour ABPM
Limited Data on Pediatric Hypertension Treatment Goals
Need for Prospective Trials on BP Management in Children with CKD
Limited Research on Pediatric-Specific Biomarkers for CKD
Insufficient Screening for Preterm Infants at Risk of CKD
Lack of Early Screening and Intervention for CKMS in Children
**Tertiary Prevention**
Limited International and Long-term Pediatric CKD Cohort Data
No Consensus on Preferred HRQOL Assessment Tools
Inadequate Resources and Trained Personnel for Pediatric Dialysis in Some Regions
Technical Challenges in Dialysis for Neonates and Small Children
Lack of Consensus on Optimal Timing for Pediatric Kidney Transplantation
Insufficient Research on Risks Associated with Pediatric Kidney Transplantation

## Data Availability

Data are contained within the article.
